# Long-Term Functional Outcomes After Sepsis for Adult and Pediatric Critical Care Patients—Protocol for a Systematic Review

**DOI:** 10.3389/fped.2021.734205

**Published:** 2021-10-25

**Authors:** Adam Simpson, Deborah Long, Carolin Fleischmann-Struzek, Jessicah Minogue, Balasubramanian Venkatesh, Naomi E. Hammond, David H. Tian, Luregn J. Schlapbach

**Affiliations:** ^1^Child Health Research Center, The University of Queensland, Brisbane, QLD, Australia; ^2^Pediatric Intensive Care Unit, Queensland Children‘s Hospital, Brisbane, QLD, Australia; ^3^Center for Healthcare Transformation, Queensland University of Technology, Brisbane, QLD, Australia; ^4^Institute of Infectious Diseases and Infection Control, Jena University Hospital, Jena, Germany; ^5^Center for Sepsis Control and Care, Jena University Hospital, Jena, Germany; ^6^The Wesley and Princess Alexandra Hospitals, Brisbane, QLD, Australia; ^7^The George Institute for Global Health and University of New South Wales Sydney, Sydney, NSW, Australia; ^8^Department of Intensive Care, Royal North Shore Hospital, Sydney, NSW, Australia; ^9^Department of Anesthesia and Perioperative Medicine, Westmead Hospital, Sydney, NSW, Australia; ^10^Department of Intensive Care and Neonatology, and Children‘s Research Center, University Children‘s Hospital Zurich, Zurich, Switzerland

**Keywords:** sepsis, septic shock, quality of life, long-term outcome, child, adult, survivorship

## Abstract

**Objective:** Sepsis is responsible for a massive burden of disease, with a global estimate of 48.9 million cases resulting in approximately 11 million deaths annually. Survivors of sepsis may also experience long-term impairments that can persist for years after hospital discharge. These cognitive, physical and/or psychosocial deficits may contribute to a lower health related quality of life and represent a significant ongoing burden to the individual, the community and the health care system. We aim to systematically review the available evidence on long-term functional and quality of life outcomes after sepsis in children and adults.

**Data Sources:** Medline, EMBASE, and CINAHL will be searched for eligible studies.

**Study Selection:** Studies of adult and pediatric survivors of sepsis who had required admission to intensive care will be included. A minimum 6 month prospective follow up will be required. Accepted outcomes will be any validated measure of health-related quality of life (HRQoL) or functional deficits, using the Post-Intensive Care Syndrome (PICS) framework of cognitive, physical or psychosocial outcomes.

**Data Extraction:** Data extraction will include information related to study characteristics, population characteristics, clinical criteria and outcomes.

**Data Synthesis:** Studies meeting the inclusion criteria will be presented descriptively separated for pediatric and adult age groups. Meta-analysis will be attempted if sufficient primary data from several studies applying the same tests and outcomes are available. The primary outcome is HRQoL after sepsis; secondary outcomes include the functional status at follow-up.

**Conclusions:** This systematic review will define the long-term impact of sepsis survivorship. The data will contribute to informing patient, clinician and stakeholder decisions and guide further research and resource management.

## Introduction

Sepsis is defined as a “life-threatening organ dysfunction caused by a dysregulated host response to infection” (1, 2). Septic shock refers to a severe subtype of sepsis involving “profound circulatory, cellular, and metabolic abnormalities” with an associated higher mortality. Sepsis represents a massive global health burden with an estimated 48.9 million cases resulting in 11 million deaths annually (3–5). In 2017, the WHO identified sepsis as a global health priority (6, 7). Subsequent to the WHO resolution, several healthcare systems have invested in action plans to implement measures for the prevention, diagnosis, management, and follow-up of sepsis (8).

In adults admitted to intensive care, sepsis-related mortality has decreased over the past decades from 35.0 to 18.4% in a large epidemiological study in Australia and New Zealand (9). In critically ill children, the decline in sepsis mortality paralleled the decrease in mortality observed in other diseases and is currently around 9% (10, 11). In 2017, a large epidemiological study in the US of patients with sepsis observed that sepsis incidence using clinical criteria remained stable over the 2009 to 2014 period, and that sepsis was present on average in 6% of adult hospitalizations, making it one of the most common conditions (12). The cost of sepsis has been estimated to USD$16,000 per admission for sepsis, and USD$ 38,000 for septic shock (13). The annual cost of sepsis to the US health care system is estimated at USD$24 billion, or 13% of total hospital expenditure whilst accounting for 3.6% of hospital admissions. Importantly, these figures only represent the short-term burden of sepsis.

Survivors of critical illness often suffer long-term consequences including poor health-related quality of life (HRQoL) and new functional impairments that may persist for years after hospital discharge (14, 15). These deficits can occur across cognitive, physical and psychosocial domains and are often referred to as Post-Intensive Care Syndrome (PICS) (16). PICS provides a framework to facilitate a better understanding and promote consistent reporting of long-term outcomes after critical illness. Survivors of sepsis, however, may be at particularly high risk of developing long term sequalae and the term “Post-Sepsis Syndrome” (PSS) has been proposed to reflect the unique pathophysiological insults associated with sepsis (17–19). A systematic review from 2009 by Winters et al., demonstrated that survivors of sepsis suffered worse HRQoL compared to non-septic, critically ill and community controls (20). A landmark study by Iwashyna et al. in 2010 found the rate of moderate to severe cognitive impairment increased almost three-fold from 6.1 to 16.7% after an episode of sepsis (21). It also demonstrated a significant increase in difficulties associated with activities of daily living. Cognitive impairment after sepsis includes deficits in memory, attention, language and executive function (22). Mechanisms include impaired brain oxygen delivery, disruption of the blood-brain barrier, neuroinflammation and neurotransmitter imbalance (23). Prolonged immobility combined with inflammatory damage to nerves and muscles contributes to ICU acquired weakness and physical disability (24). Septic patients also have longer average ICU length of stay and thus increased exposure to the general insults inherent to intensive care (13). These additional exposures may lead to important differences in long-term sequelae between survivors of septic and non-septic critical illness. Finally, mental health impacts such as depression, anxiety and post-traumatic stress disorder are all frequently reported following critical illness and sepsis (18, 19).

While the impact of sepsis on long-term outcomes has been widely recognized for adult and neonatal age groups, the relevance for children is becoming increasingly noticed in view of the major impact of sepsis on child health outcomes (5, 25). The pediatric population may be subject to different epidemiology, treatments, and susceptibility to injury resulting in age-specific long-term outcomes (26, 27). Due to these important differences, a separate pediatric PICS frame-work (PICS-p) has been proposed, and pediatric Core Outcome Sets (COS) have been established (28–30).

There is thus a need for contemporary data providing an estimate on the impact of sepsis on health-related quality of life and functional outcomes across adult and pediatric age groups. We therefore aim to systematically review the literature on hrQoL and functional status in patients admitted to intensive care with sepsis, published in the past decade. Specifically, we aim to describe the prevalence of survival with reduced hrQoL and functional outcomes in sepsis survivors in a meta-analysis, to assess changes over time during recovery, and to compare the outcomes with other critically ill patients, populational reference groups, and baseline data, where available.

## Methods

A systematic review of long-term health related quality of life (HRQoL) and functional impairment after sepsis in adult and pediatric patients will be conducted. It follows the Preferred Reporting Items for Systematic Review and Meta-Analysis Protocols statement 2020 (PRISMA) (31). The study protocol has been registered in the international prospective register of systematic reviews (28 April, 2020; PROSPERO–CRD42020164309) and the protocol follows PRIMSA-P (32) recommendations.

### Participants

Studies on adults or children diagnosed with sepsis, admitted to an intensive care unit and with follow-up data at least 180 days after sepsis diagnosis will be included. Follow up data must include information on the participant‘s health related quality of life and/or functional status as outlined below. Adults will be defined as equal or >18 years old. Children will be defined as older than 28 days adjusted age (thus excluding neonates) to <18 years old.

Our definition of sepsis had to consider the changing definitions of sepsis over the past decade. In 2001 the ACCP/SCCM Consensus Conference and International Sepsis Definition Conference (sepsis-1/2) defined sepsis as the presence of infection and Systemic Inflammatory Response Syndrome (SIRS) criteria, severe sepsis as sepsis and new organ dysfunction, and septic shock as sepsis with refractory hypotension (23). An updated consensus definition established in 2016 (sepsis-3) removed the SIRS criteria and defined sepsis as infection and new organ dysfunction, as defined by an increase of two or more points in the Sequential Organ Failure Assessment (SOFA) score. Septic shock was defined as sepsis with refractory hypotension and a serum lactate >2 mmol/L (1). In order to capture studies using both criteria we have defined sepsis as “infection with organ dysfunction”. This will encapsulate severe sepsis or septic shock (as per sepsis-1/2 criteria) in adults (23), severe sepsis or septic shock in children (24) (as per 2005 Goldstein criteria) and sepsis or septic shock (as per sepsis-3) in adults (1). We will be collectively referring to these concepts as “sepsis.”

Studies will be limited to those reporting on patients admitted to an adult Intensive Care Unit (ICU) or a pediatric ICU (PICU), because most patients with severe organ dysfunction are expected to be managed in ICU during their stay in settings where ICU facilities are available.

### Intervention

Not applicable. The purpose is to review studies describing outcomes in patients with sepsis.

### Comparison

Where available, we will compare outcomes of ICU survivors with sepsis with non-sepsis critically ill patients and community controls; as well as with their pre-sepsis HRQoL and functional status, if recorded.

### Outcomes

The primary outcome is Health Related Quality of Life (HRQoL) at 6 to <12 months after diagnosis of sepsis.

HRQoL represents a pragmatic outcome measure that assesses multiple domains and creates a cumulative score to represent global functionality and quality of life (33). Most scores include sub-scores for different domains including physical, mental or social function. We will include any validated tool that includes a cumulative score for HRQoL, such as EQ-5D, SF-36 or PedsQoL for example (34–36).

The follow up time of at least 6 months is chosen as a pragmatic lower threshold to identify deficits which have a higher likelihood to result in persistent impact. This is based on evidence suggesting that after the initial insult of critical illness, some survivors will experience improvement back toward a new baseline that may slowly improve for months (37).

Secondary Outcomes: HRQoL at 12 to <24, and ≥24 months after sepsis, sub-domains of the HRQoL scores (e.g., physical role, social functioning) as well as functional data across the domains of the PICS framework at 6 to <12, 12 to <24, and ≥24 months after sepsis. These include measures of cognitive, physical and psychosocial outcome as well as the activities of daily living. These are aligned with the proposal to work toward universally accepted core outcome measures for critically ill adults and children (30, 38). We anticipate that multiple outcome tools will be utilized and that data will be heterogeneous.

### Risk Factors and Interventions

We will not specifically search for risk factors or interventions associated with post-sepsis outcomes. However, if such data exists in the included papers then we shall provide a descriptive review.

### Search Strategy

In order to formulate an appropriate search strategy to answer the research question: “Do adults and children diagnosed with sepsis or septic shock suffer poor long-term health related quality of life or functional outcomes”? we conducted a search around the following terms; sepsis/septic shock AND ICU/PICU AND (HRQoL OR cognitive, physical or psychological impairments). The review was registered online before its initiation (https://www.crd.york.ac.uk/PROSPERO/display_record.php?RecordID=164309). The detailed search strategy is provided as Table 1.

We will search the following databases:
Pubmed/medline
CINAHL
EMBASE.

**Table 1:** Search strategy.

***PubMed:***
((((((((((((((“Intensive Care Units” [Mesh:NoExp]) OR “Intensive Care Units” [tiab]) OR “Intensive Care Unit” [tiab]) OR “Intensive Care Units, Pediatric” [Mesh:NoExp]) OR ICU [tiab]) OR PICU [tiab]) OR “intensive care” [tiab]) OR “critical care” [tiab]) OR critical illness * [tiab]))) OR (“post-intensive care syndrome” [tiab] OR PICS [tiab]))) OR “post sepsis syndrome” [tiab])
AND
(((((“Sepsis”[Majr:NoExp]) OR “Shock, Septic”[Mesh]) OR sepsis[tiab]) OR septic[tiab]) OR septicemia[tiab])
AND
((((((((((((“Mental Disorders” [Mesh]) OR “mental illness” [tiab]) OR depression [tiab]) OR anxiety [tiab]) OR ((PTSD [tiab] OR “post-traumatic stress” [tiab])))) OR (((social function * [tiab]) OR social-impair * [tiab]) OR social-dysfunction * [tiab])) OR (((emotional-impair * [tiab]) OR emotional-dysfunction * [tiab]) OR emotional-disorder * [tiab]) OR behavior [mesh])))
OR
((((((((((((((((“Activities of Daily Living” [Mesh]) OR “Activities of Daily Living” [tiab]) OR “ADL” [tiab]) OR physical-dysfunction * [tiab]) OR physical-disorder * [tiab]) OR physical-impairment * [tiab]) OR motor-impairment * [tiab]) OR motor-dysfunction * [tiab]) OR motor-disorder * [tiab]) OR ICUAW [tiab]) OR ICU-AW [tiab]) OR neuromyopathy [tiab]) OR neuropathy [tiab]) OR myopathy [tiab])))
OR
((((((((“Cognitive Dysfunction” [Mesh]) OR “Cognitive Dysfunction” [tiab]) OR “Cognitive Dysfunctions” [tiab]) OR Cognitive-impairment * [tiab]) OR Neurocognitive-impairment * [tiab]) OR Neurocognitive-disorder * [tiab]) OR Neurocognitive-dysfunction * [tiab]))) OR (((((((“Quality of Life” [Mesh]) OR “Quality of Life” [tiab]) OR “Health Related Quality Of Life” [tiab]) OR HRQoL [tiab]) OR HRQoL [tiab]) OR QoL [tiab]))
***Embase***
“intensive care unit”/exp OR “Intensive Care Units”:ti,ab OR “Intensive Care Unit”:ti,ab OR “pediatric intensive care unit”/exp OR ICU:ti,ab OR PICU:ti,ab OR “intensive care”:ti,ab OR “critical care”:ti,ab OR critical illness * :ti,ab OR “post-intensive care syndrome”:ti,ab OR PICS:ti,ab OR “post sepsis syndrome”:ti,ab
AND
“sepsis”/de OR “septic shock”/exp OR sepsis:ti,ab OR septic:ti,ab OR septicemia:ti,ab
AND
“mental disease”/exp OR “mental illness”:ti,ab OR depression:ti,ab OR anxiety:ti,ab OR PTSD:ti,ab OR “post-traumatic stress”:ti,ab OR “social function*”:ti,ab OR social-impair*:ti,ab OR social-dysfunction*:ti,ab OR emotional-impair * :ti,ab OR emotional-dysfunction*:ti,ab OR emotional-disorder*:ti,ab OR “behavior”/exp
OR
“daily life activity”/exp OR “Activities of Daily Living”:ti,ab OR “ADL”:ti,ab OR physical-dysfunction*:ti,ab OR physical-disorder*:ti,ab OR physical-impairment*:ti,ab OR motor-impairment*:ti,ab OR motor-dysfunction*:ti,ab OR motor-disorder*:ti,ab OR ICUAW:ti,ab OR ICU-AW:ti,ab OR neuromyopathy:ti,ab OR neuropathy:ti,ab OR myopathy:ti,ab
OR
“cognitive defect”/de OR “Cognitive Dysfunction”:ti,ab OR “Cognitive Dysfunctions”:ti,ab OR Cognitive-impairment*:ti,ab OR Neurocognitive-impairment*:ti,ab OR Neurocognitive-disorder*:ti,ab OR Neurocognitive-dysfunction*:ti,ab OR “mild cognitive impairment'/exp OR “quality of life”/exp OR “Quality of Life”:ti,ab OR “Health Related Quality Of Life”:ti,ab OR HRQoL:ti,ab OR HRQoL:ti,ab OR QoL:ti,ab
AND
[embase]/lim AND ([article]/lim OR [article in press]/lim) AND [english]/lim
***CINAHL:***
(MH “Intensive Care Units”) OR (MH “Intensive Care Units, Pediatric”) OR “Intensive Care Units” OR “Intensive Care Unit” OR ICU OR PICU OR “intensive care” OR ”critical care” OR critical illness* OR “post-intensive care syndrome” OR PICS OR “post sepsis syndrome”
AND
MH “Sepsis” OR MH “Shock, Septic” OR sepsis OR septic OR septicemia
AND
MH “Mental Disorders+” OR “mental illness” OR depression OR anxiety OR PTSD OR “post-traumatic stress” OR social function* OR social-impair* OR social-dysfunction* OR emotional-impair* OR emotional-dysfunction* OR emotional-disorder* OR MH “Behavior+”
OR
MH “Activities of Daily Living+” OR “Activities of Daily Living” OR “ADL” OR physical-dysfunction* OR physical-disorder* OR physical-impairment* OR motor-impairment* OR motor-dysfunction* OR motor-disorder* OR ICUAW OR ICU-AW OR neuromyopathy OR neuropathy OR myopathy
OR
MH “Cognition Disorders+” OR “Cognitive Dysfunction” OR “Cognitive Dysfunctions” OR Cognitive-impairment* OR Neurocognitive-impairment* OR Neurocognitive-disorder* OR Neurocognitive-dysfunction* OR (MH “Quality of Life+“) OR “Quality of Life” OR “Health Related Quality Of Life” OR HRQoL OR HRQoL OR QoL

### Study Selection Criteria

#### Inclusion

Studies will be included if they meet the following criteria:

(1) Subjects diagnosed with sepsis or septic shock operationalized as infection with organ dysfunction. This will include severe sepsis or septic shock (sepsis-1/2) in adults, severe sepsis or septic shock in children and sepsis or septic shock (sepsis-3) in adults (1, 39, 40). For the purposes of this protocol the above shall be referred to as “sepsis.”
(2) Admitted to an ICU or PICU.
(3) Underwent follow up at least 180 days post sepsis diagnosis.
(4) Follow up includes a validated measure of health-related quality of life and/or functional assessment. Functional domains will be cognitive, physical, or psychosocial deficits or function deficits in the activities of daily living as per the PICS framework.
(5) Age >28 days old (>44 weeks corrected gestational age in preterm infants) at time of sepsis.
(6) Studies reporting on original data including prospective observational studies and randomized-controlled trials. Patients may be retrospectively recruited (i.e., from databases) but must have prospective longitudinal follow up.
(7) Outcome data must be available for at least 20 survivors of sepsis.
(8) Publication in English.
(9) Studies published between January 2009 to March 2021 in order to capture more contemporary data. Additionally, more recent studies that the authors have complete access to will be considered.

Trials reporting on >180 day HRQOL or functional outcomes in other populations (general intensive care population) will be eligible if their publication provides separate information specific to survivors of sepsis.

#### Exclusion

We will exclude studies on long-term outcome in neonatal and premature neonatal patients; studies reporting on mortality alone; gray literature (unpublished data); conference notes and abstracts; studies not published in English; and studies not providing original data (such as reviews or opinion papers).

### Study Selection

Study screening will be done with the aid of the web-based systematic review software COVIDENCE. Studies will be independently reviewed and unanimously selected by two investigators. Disagreement will be solved by referral to a third reviewer. Initially title and abstract will be screened in relation to suitability for the review. The papers identified will then have the full text assessed by two investigators using the same approach as above. Eligibility will be assessed using the above inclusion and exclusion criteria. Multiple studies on the same patient cohort will be reviewed. If they report different outcome data they will be included. Re-analysis of previously published results will be excluded. If required authors will be contacted for clarification. Selected articles will then be subjected to quality assessment and data extraction. Selected papers will also have their reference list reviewed by a single reviewer for potentially relevant papers based on title alone. Relevant papers will be added for full text review as outlined above. A PRISMA flow diagram will be provided.

### Data Extraction

Two independent reviewers will extract data using a pre-determined form. Information will be gathered on study characteristics (author, country/countries of origin, year of publication, study design), population characteristics (adult or pediatric, age and gender distribution, patient numbers, comorbidities, disease severity) and outcomes (duration of follow up, survival to follow up, loss to follow up, type of follow-up measures, and incidence of each outcome measure of interest as above). Only data published within the text or supplements will be considered, unpublished data will be excluded.

### Confidence Assessment

Two independent reviewers will assess each selected paper for bias using the Newcastle-Ottawa Scale (NOS) (41). Disagreement will be resolved by consensus or a third independent reviewer. NOS utilizes a “star system”: “Stars” are attributed to study characteristics deemed to contribute to a lower risk of bias with a total of nine available across the three domains of patient selection, comparability and outcome. A summative score of 6 or more indicates low risk of bias, 4–5 indicates moderate risk and three or less a high risk. Septic cohorts from RCTs will also be assessed as cohorts using NOS rather than a specific RCT tool. This is justified as we are interested in longitudinal outcomes rather than the effect of interventions. A pre-determined assessment form will be used. No study will be excluded from the synthesis based on study quality assessment.

### Analysis Plan

In the first instance we intend to provide a descriptive analysis of the results. This will be undertaken using the structure set out by the Economic and Social Research Council (ESRC) methods programme (42). This will include preliminary description and synthesis of the findings, exploring relationships in the data, and assessing the robustness of the synthesis.

We expect outcome data to be highly variable with multiple outcome tools used. Therefore, we will classify studies with respect to

- outcome measurement tool/score reported
- observation time (in months post-sepsis: 6 to <12 months, 12 to <24 months, ≥24 months)
- pediatric (up to <18 years) and adult age groups (≥18 years). Papers primarily reporting on adult patients with occasional representation of older adolescents (>14 to <18 years) will be analyzed as adult papers.

Where there are few studies reporting on the same outcome measure, attempts will be made to broaden the categorization across wider post-sepsis observation times to facilitate more robust statistical analysis. The primary outcome analyzed will be the total score for health-related quality of life. Analysis will be performed whereby appropriately presented SF-36 outcomes will be converted to mean EQ-5D score using the techniques of Ara and colleagues, so as to increase the statistical power of the analysis (43). The secondary outcomes will focus on the respective sub-domains of the HRQoL scores and domains relating to functional status as per the PICS framework. Standardized mean difference with Cohen's d will also be used to permit aggregation of different measurement metrics. The effect sizes will be interpreted using conventional criteria (0.2 = small, 0.5 = medium, 0.8 = large).

Meta-analysis of means using a fixed- or random-effects model will be utilized, depending on the degree of heterogeneity identified. Heterogeneity will be assessed using the *I*^2^ statistic, where *I*^2^ < 25%, between 25 and 50%, between 50 and 75%, and >75% represent low, moderate, substantial, and considerable heterogeneity, respectively. Where substantial or considerate heterogeneity is encountered, a Baujat plot will be used to identify studies with high contributions to heterogeneity. Leave-one-out analyses will be performed to identify potential influential studies that produced a large change in the pooled estimates after they were left out one at a time from all studies.

Evidence of publication bias will be sought using the methods of Egger et al. (44) and Begg et al. (45), as well as the use of contour-enhanced funnel plots. All P values will be two-sided, with significance determined at <0.05. Statistical analyses will be performed in R (version 4.0.2, R Foundation for Statistical Computing, Vienna, Austria).

### Significance

Of the estimated 37 million annual survivors of sepsis, up to 50% may suffer some form of persistent deficit (3, 19). This translates into a massive burden of chronic disease for the patients, families, and the community, being described as a “hidden public health disaster” (46). Importantly, the burden of chronic illness in survivors of sepsis is expected to worsen as the number of survivors increases (9), implying an urgent need to systematically review the current available evidence on long-term outcomes after sepsis. Previous systematic reviews (20, 47) have helped bring attention to these issues but are based on aging data, have a more narrow focus on mortality and quality of life outcomes, and only include adult populations. This systematic review of long-term quality of life and functional outcomes after sepsis in adults and children will include data reported in the literature across the age groups from the last decade, thus maintaining a focus on more contemporary practice.

We opt to restrict the survey to publications from 2009 onwards for several reasons: First, the publication of the Surviving Sepsis Campaign in 2008 (48) was a sentinel event for sepsis care and resulted in wide uptake. Second, the epidemiology of sepsis changed substantially in most geographical regions with a shift from meningococcal sepsis to other pathogens (11). Third, fundamental critical care treatment approaches developed substantially during 1990–2008 with the establishment of interventions such as early goal directed therapy or low tidal volume ventilation, and optimal glucose management, and the time from 2009 onwards is more likely comparable to current practice.

Specifically, we plan to assess the incidence, type and severity of identified deficits. Aligned with the PICS framework, we will assess both overall hrQoL, as well as it subdomains, and the domains pertinent to functional status. While this review strategy is anticipated to yield a more comprehensive picture of the entire burden on sepsis survivorship, we anticipate a number of challenges: Due to a range of available follow-up tests, heterogeneity of the used follow-up measures is likely. We aim to address this challenge by describing study results for all studies meeting inclusion criteria, while restricting meta-analyses to studies providing SF-36 and/or EQ-5D outcomes. Furthermore, while we expect that studies will use different follow-up durations, we have set the minimum at 6 months to capture follow-up beyond the acute rehabilitation phase. Finally, studies may apply different methods in terms of comparison groups, and only studies with a pre-sepsis baseline may be able to reliably distinguish sepsis-related effects from effects related to underlying comorbidities.

The review is expected to provide the most contemporary estimate of the long-term burden of sepsis in adults and children. The findings are anticipated to inform stakeholders, clinicians, and researchers aiming to address one of four key postulates of the WHO resolution on sepsis–improving survivorship and post-sepsis care for patients and families (6).

## Author Contributions

LS and AS designed the study with contribution from all authors and wrote the first draft of the manuscript. All authors contributed to abstract and fulltext review, data extraction, contributed to manuscript writing, and approved the final version of the manuscript.

## Funding

BV was supported by an MRFF Practitioner Fellowship. NH was supported by an NHMRC Investigator Fellowship.

## Conflict of Interest

The authors declare that the research was conducted in the absence of any commercial or financial relationships that could be construed as a potential conflict of interest.

## Publisher's Note

All claims expressed in this article are solely those of the authors and do not necessarily represent those of their affiliated organizations, or those of the publisher, the editors and the reviewers. Any product that may be evaluated in this article, or claim that may be made by its manufacturer, is not guaranteed or endorsed by the publisher.
